# Mammalian‐enabled (MENA) protein enhances oncogenic potential and cancer stem cell‐like phenotype in hepatocellular carcinoma cells

**DOI:** 10.1002/2211-5463.12254

**Published:** 2017-06-22

**Authors:** Kunpeng Hu, Pinzhu Huang, Hui Luo, Zhicheng Yao, Qingliang Wang, Zhiyong Xiong, Jizong Lin, He Huang, Shilei Xu, Peng Zhang, Bo Liu

**Affiliations:** ^1^ Department of General Surgery The Third Affiliated Hospital Sun Yat‐sen University Guangzhou China; ^2^ Department of Gastrointestinal Surgery The Sixth Affiliated Hospital Sun Yat‐sen University Guangzhou China; ^3^ Department of Operating Room The Third Affiliated Hospital Sun Yat‐sen University Guangzhou China

**Keywords:** hepatic cancer stem cell, hepatic progenitor markers, hepatocellular carcinoma, mammalian‐enabled, tumorigenicity

## Abstract

Mammalian‐enabled (MENA) protein is an actin‐regulatory protein that influences cell motility and adhesion. It is known to play a role in tumorigenicity of hepatocellular carcinoma (HCC) but the underlying molecular mechanism remains unknown. This study aimed to investigate the oncogenic potential of MENA and its capacity to regulate cancer stem cell (CSC)‐like phenotypes in HCC cells. Real‐time‐PCR and western blot were used to assess mRNA and protein levels of target genes in human HCC tissue specimens and HCC cell lines, respectively. Stable MENA‐overexpressing HCC cells were generated from HCC cell lines. Transwell cell migration and colony formation assays were employed to evaluate tumorigenicity. Ectopic expression of MENA significantly enhanced cell migration and colony‐forming ability in HCC cells. Overexpression of MENA upregulated several hepatic progenitor/stem cell markers in HCC cells. A high MENA protein level was associated with high mRNA levels of MENA, CD133, cytokeratin 19 (CK19), and epithelial cell adhesion molecule (EpCAM) in human HCC tissues. Overexpression of MENA enhanced epithelial‐to‐mesenchymal transition (EMT) markers, extracellular signal‐regulated kinases (ERK) phosphorylation, and the level of β‐catenin in HCC cells. This study demonstrated that overexpression of MENA in HCC cells promoted stem cell markers, EMT markers, and tumorigenicity. These effects may involve, at least partially, the ERK and β‐catenin signaling pathways.

**Abbreviations**
ChIP: chromatin immunoprecipitation
CK19: cytokeratin‐19
CSCs: cancer stem cells
DFS: disease‐free survival
EMT: epithelial‐to‐mesenchymal transition
EpCAM: epithelial cell adhesion molecule
HCC: hepatocellular carcinoma
HPC: hepatic progenitor cell
LCSCs: liver CSCs
MENA: mammalian‐enabled
SEM: standard error of the mean

Liver cancer is one of the most common malignant tumors, with a worldwide incidence ranked No. 5 and a mortality rate ranked No. 3 in all malignant tumors 1. In 2012, there are an estimated of 782 500 new cases and 745 500 deaths related to liver cancer worldwide 2. In particular, China has a high incidence of liver cancer, accounting for about 50% of the global cases 2. Hepatocellular carcinoma (HCC) is the most common subtype of liver cancer, accounting for about 85–90% of all primary liver cancers 3, with a poor prognosis due to the high rates of recurrence and metastasis 4.

Growing evidence suggests that cancer stem cells (CSCs) play an important role in the tumorigenicity of a tumor 5. According to the CSCs theory, CSCs are defined as a rare subset of cancerous cells within a tumor which are capable of self‐renewal and regulating formation and development of tumor 6. CSCs have been isolated in a variety of cancers, including HCC 7, breast cancer 8, pancreatic cancer 9, colon cancer 10, and melanoma 11. Liver CSCs (LCSCs) have been shown to possess significant capacities of migration and invasion and resistant to radiation and chemotherapy, thus contributing to the tumor recurrence and metastasis 12. Therefore, studying the molecular mechanisms underlying the role of LCSCs in recurrence and metastasis may be helpful to find new treatment strategies for liver cancer. Currently, several LCSC‐related genes and biomarkers have been identified. It has been shown that human HCC cells with CD133 surface marker have the self‐renewal capacity, and marked proliferation and colony formation potentials *in vitro*, as well as a remarkable tumorigenicity *in vivo* 13. Cytokeratin‐19 (CK19) is a LCSCs marker significantly associated with tumor size, reduced tumor differentiation, invasion, and metastasis 14, 15. In addition, the epithelial‐to‐mesenchymal transition (EMT)‐related proteins were upregulated in CK19^+^ HCC cells 16. Epithelial cell adhesion molecule (EpCAM) is a tumor‐associated cell adhesion molecule with oncogenic potential in many endothelium‐derived cancers 17. Terris *et al*. 18 have demonstrated that EpCAM^+^ HCC cells have the characteristics of hepatic stem/progenitor cells and are able to form a highly invasive tumor in immunodeficient mice. Kimura *et al*. 19 showed that EpCAM^+^ HCC cells have a greater ability for colony formation *in vitro* and tumor formation *in vivo* as compared with EpCAM^‐^ HCC cells. Studies have shown that HCC cells with surface markers CD133, CD90, CD44, CK19, and EpCAM possess LCSC‐like characteristics 20, 21, 22. All these findings suggest that HCC cells expressing CSC biomarkers exhibit the characteristic of LCSCs and have a stronger tumorigenicity. However, the molecular mechanism about the regulation of expression of LCSC‐related biomarkers remains not fully understood. Mammalian‐enabled (MENA) is an actin‐regulatory protein with a molecular weight of 80 kD and has the functions such as cell motility and adhesion 23. MENA is undetectable in many normal tissues, but is highly expressed in gastric cancer, breast cancer, cervical cancer, colorectal cancer, pancreatic cancer, salivary gland cancer, and other adenocarcinomas; thus, it could be used as a tumor marker for these cancers 23. In addition, researches on breast cancer have shown that expression of MENA is associated with tumor invasion and metastasis 24. In the studies on hepatocellular carcinoma 25, MENA may be involved in the development and progression of tumors. Our previous study on 81 patients with HCC found that MENA is overexpressed in 40.74% paraffin‐embedded HCC specimens. Compared to MENA‐negative control, poor cellular differentiation, advanced tumor stage, and worse disease‐free survival (DFS) have been found in MENA‐positive group. Furthermore, multivariate Cox regression analysis shows that MENA overexpression is a risk factor for DFS (HR: 2.309, 95% CI: 1.104–4.828; *P* = 0.026) 26. Recently, Lin *et al*. 25 demonstrated that knockdown of MENA reduced HCC cell migration and invasion *in vitro*, as well as their metastasis capacity *in vivo*. These results suggest an oncogenic potential for MENA.

Even though the oncogenic potential of MENA in HCC cells has been revealed, however, the underlying molecular mechanism remains to be investigated. MENA is an EMT‐dependent alternatively spliced gene. The splice isoform of MENA with the exon 11a is a characteristic for epithelial cells and is not found in mesenchymal cells 27. Meanwhile, the splice isoform of MENA has also been found to be expressed in primary tumor cells, but not in invasive tumor cells 28. Although the detailed mechanism is still unknown, these findings suggest that *MENA* gene may play a role in the regulation of EMT. Multiple signaling pathways have been shown to be involved in the regulation of EMT and CSC transition 29, 30, and there are many common regulation mechanisms between EMT and CSC. For instance, extracellular signal‐regulated kinase (ERK) signaling has been shown to be involved in the regulation of both stemness 31 and EMT 32 in several cancers. Wnt/β‐catenin pathway can promote the expression of surface markers of liver cancer and the promotion of liver CSC activation 33 and is involved in EMT of HCC 34. Based on these observations, we hypothesized that MENA may play a role in the regulations of CSC and EMT in HCC cells. The purpose of this study was to investigate the oncogenic potential of MENA and its capacity to regulate CSC and EMT phenotypes in HCC cells. By using HCC tumor tissue samples and cancer cell lines, the above issues were investigated.

## Materials and methods

### HCC samples

A total of 81 tissue specimens of HCC were collected from primary HCC patients undergoing curative surgery in our center between March 2010 and July 2012 as previously described 26. The median age of the patients was 49 years (range: 13–80 years); the median tumor size was 4.3 cm (range: 1.5–10 cm). All the patients were diagnosed with primary HCC; 69 (85%) patients were diagnosed with chronic viral hepatitis (HBV: 66 patients and HCV: three patients). This study was approved by the Institutional Review Board of the Third Affiliated Hospital of Sun Yat‐sen University. Written informed consent was obtained from all the patients.

### Cell culture

Hepatocarcinoma cell (HCC) lines QGY‐7703 and PLC‐8024 were obtained from the Institute of Virology, Chinese Academy of Medical Sciences (Beijing, China), while SMMC‐7721, BEL‐7402, HUH‐7, MHCC‐97L, and MHCC‐97H were obtained from Liver Cancer Institute of Fudan University (Shanghai, China). All the hepatocarcinoma cell (HCC) lines were cultured by continuous passage in Dulbecco's modified Eagle medium (Gibco, Carlsbad, CA, USA) supplemented with 10% fetal bovine serum (Gibco) and 1% penicillin/streptomycin (Gibco). Cells were maintained in a humidified incubator at 37 °C in 5% CO_2_. RNA and protein were extracted from exponentially growing cells.

### Generating stable MENA‐overexpressing HCC cell lines

For stable overexpression of MENA, SMMC‐7721 and QGY‐7703 cells were infected with pLVX‐IRES‐Puro‐MENA viral particles (Clontech; Mountain View, CA, USA) and selected by puromycin according to the manufacturer's protocol. The established stable cell lines were designated as SMMC‐7721‐MENA and QGY‐7703‐MENA, respectively.

### Western blot

Total proteins were extracted with RIPA lysis buffer and separated by SDS/PAGE and then transferred to the poly(vinylidene difluoride) membranes. The membranes were blocked with 5% skimmed milk and incubated with the appropriate antibody. The antigen–antibody complex on the membrane was detected with enhanced chemiluminescence regents (Thermo Scientific, Rockford, IL, USA). The antibodies including ERK (Cat.#9926, Cell Signaling, Danvers, MA, USA) and p‐ERK (Thr202/Tyr204, Cat.#9910, Cell signaling), E‐cadherin (Cat.#sc‐7870, Santa Cruz, Santa Cruz, CA, USA), vimentin (Cat.#sc‐6260, Santa Cruz), β‐catenin (Cat.# 9562, Cell signaling), MENA (Cat.# 2075, Cell signaling), and α‐tubulin (Cat.#ab15246, Abcam, Cambridge, MA, USA) were all used in this study.

### *In vitro* cell migration assay

*In vitro* cell migration assays were performed in Transwell chambers (8 μm pore size; Costar). The Transwells were put into the 24‐well plates. A total of 2 × 10^4^ cells were placed into the top chamber of each insert (BD Biosciences, Eugene, OR, USA) and incubated at 37 °C for 48 h. The migrated cells were fixed with 4% paraformaldehyde for 5 min, stained with 1% crystal violet for 30 s, and then quantified by counting cells at 20× magnification (five fields per chamber).

### Colony formation assay and soft agar colony formation assay

For colony formation assay, QGY‐7703‐MENA and QGY‐7703‐Vector cells (200 cells/well) were trypsinized, plated on six‐well plates, and then cultured in medium containing 10% FBS for 2 weeks. The colonies were fixed with 4% paraformaldehyde for 5 min and stained with 1% crystal violet for 30 s. To evaluate anchorage‐independent cell growth, an assay of colony formation in soft agar was performed. A bottom layer was formed with 0.6% agar and Dulbecco's modified Eagle's medium (DMEM) plus 10% FBS in six‐well plates. After the bottom layer solidified, 5000 cells per plate, 0.4% agar, and DMEM plus 10% FBS were added to the top layer. After 2 weeks of incubation, colonies were fixed with 4% paraformaldehyde for 5 min, stained with 1% crystal violet for 30 s, and counted for each plate.

### RT‐PCR

Total RNA was extracted from human HCC tissues and HCC cell line using Trizol reagent (Invitrogen, Carlsbad, CA, USA) according to the manufacturer's instructions. Total RNA extracts of CD133^+^ and CD133^‐^ hepatoma cell lines (Huh7 and PLC‐8024) were kindly provided by Guan XY (Sun Yat‐sen University Cancer Center, China) 35. First‐strand cDNA was synthesized with 2 μg of total RNA using a First‐Strand Synthesis System (Invitrogen) according to the manufacturer's instructions. Real‐time (RT) PCR was performed using a CFX96 Real‐Time System (Bio‐Rad, Hercules, CA, USA). 2 9 SYBR Green Master mixture (Invitrogen) was used in a total volume of 10 μL. The PCR primer sequences were as follows: MENA: sense 5′‐GTGCCATTCCTAAAGGGTTGA‐3′, antisense 5′‐GCT GCCAAAGTTGAGACCATAC‐3′; CK19: sense 5′‐GTGC CATTCCTAAAGGGTTGA‐3′, antisense 5′‐GCTGCCAA AGTTGAGACCATAC‐3′; CD133: sense 5′‐TGGATGCAGAACTTGACAACGT‐3′, antisense 5′‐ATACCTGCTACGACAGTCGTGGT‐3′; EpCAM: sense 5′‐ATGTTTGG TGATGAAGGCAGAA‐3′, antisense 5′‐ATCGCAGTCAG GATCATAAAGC‐3′; ABCG2: sense 5′‐TCATCAGCCTCGATATTCCATCT‐3′, antisense 5′‐R GGCCCGTGGAACATAAGTCTT‐3; Oct4: sense 5′‐CGCAAGCCCTCAT TTCAC‐3′, antisense 5′‐CATCACCTCCACCACCTG‐3′; Sox2 F: TACAGCATGTCCTACTCGCAG‐3′, R: GAGGAAGAGGTAACCACAGGG‐3′; β‐catenin: sense 5′‐ACAACTGTTTTGAAAATCCA‐3′, antisense 5′‐CGAG TCATTGCATACTGTCC‐3′; GAPDH: sense 5′‐TGTTGC CATCAATGACCCC‐3′ and antisense 5′‐CTCCACGACGTACTCAGC‐3′, which was used as an internal control. All reactions were run in triplicate in three independent experiments.

### Chromatin immunoprecipitation (ChIP)

Stable MENA‐overexpressing HCC cells (QGY‐7703‐MENA) were trypsinized and treated with 1% formaldehyde for crosslinking, followed by sonication to lyse the cells and break down chromatin. The mixture of DNA and protein was immunoprecipitated with anti‐β‐catenin antibody. After extensive washing, elution, and de‐crosslinking processes, the chromatin immunoprecipitation (ChIP)‐DNA fragments were finally obtained. ChIP‐DNA fragments were used as a template for PCR with specific primer pair for the promoter region of *MENA* gene (sense 5′‐TATATGCCCCTCCTCAAC‐3′, antisense 5′‐GTTCCTC GCCCGTGGTTG‐3′).

### Statistical analysis

Statistical analyses were performed using spss 17.0 for Windows (SPSS, IBM Corp., Armonk, NY, USA) and graphpad Prism6 (GraphPad Software, La Jolla, CA, USA). Data were expressed as the mean ± standard error of the mean (SEM) from at least three independent experiments. Quantitative data between groups were compared using the Student's *t*‐test. A two‐tailed *P* value of < 0.05 was considered as statistical significance.

## Results

### Generating MENA‐overexpressing HCC cell lines

**Figure 1:**
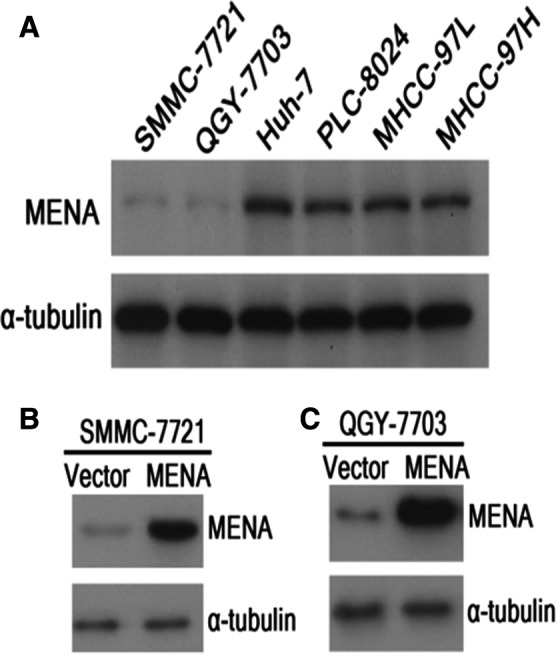
Generating MENA‐overexpressing HCC cell lines. (A) MENA protein levels of six HCC cell lines were determined by western blot. SMMC‐7721 (B) and QGY‐7703 (C) were used to generate the stable MENA‐overexpressing HCC cells. The overexpression of MENA proteins was confirmed by western blot.

Our previous work showed that overexpression of MENA is associated with poor cellular differentiation, advanced tumor stage, and worse DFS in HCC. To further investigate the oncogenic potential of MENA *in vitro*, we attempted to generate stable MENA‐overexpressing HCC cell lines. MENA protein levels were screened in six HCC cell lines. As shown in Fig. 1A, Huh‐7, PLC‐8024, MHCC‐97L, and MHCC‐97H cells were positive for MENA protein, whereas SMMC‐7721 and QGY‐7703 cells were nearly negative for MENA. Therefore, two low MENA‐expressing cell lines, SMMC‐7721 and QGY‐7703, were chosen to establish the stable MENA‐overexpressing HCC cells (SMMC‐7721 MENA and QGY‐7703 MENA). The overexpression of MENA protein levels was confirmed by western blot in SMMC‐7721‐MENA (Fig. 1B) and QGY‐7703‐MENA (Fig. 1C).

### Overexpression of MENA enhanced tumorigenicity of HCC cells

**Figure 2:**
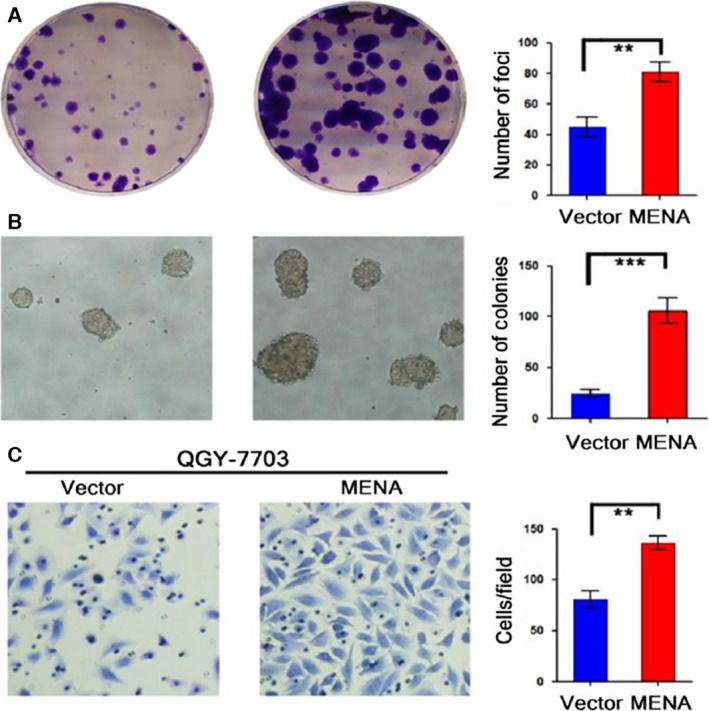
Overexpression of MENA enhanced tumorigenicity in HCC cells. Colony formation assay (A), soft agar colony formation assay (B), and transwell cell migration assay (C) were employed to determine the tumorigenicity. The results were quantified and compared between QGY‐7703‐MENA and vector control cells. **P < 0.01; ***P < 0.001.

The proliferative and metastatic capacities of MENA‐overexpressing HCC cells were determined to evaluate the oncogenic potential of MENA. As shown in Fig. 2, both colony formation (Fig. 2A, *P* < 0.001) and soft agar colony formation assay (Fig. 2B, *P* < 0.01) showed that QGY‐7703‐MENA cells can form significantly more colonies than the control cells. In addition, transwell cell migration assay showed that QGY‐7703‐MENA cells had a significantly higher migration ability as compared with the vector control cells (Fig. 2C, *P* < 0.01). These data suggested that overexpression of MENA enhanced tumorigenicity in HCC cell lines.

### Overexpression of MENA upregulated hepatic progenitor/stem cell markers and Wnt/β‐catenin signaling markers in HCC cells

**Figure 3:**
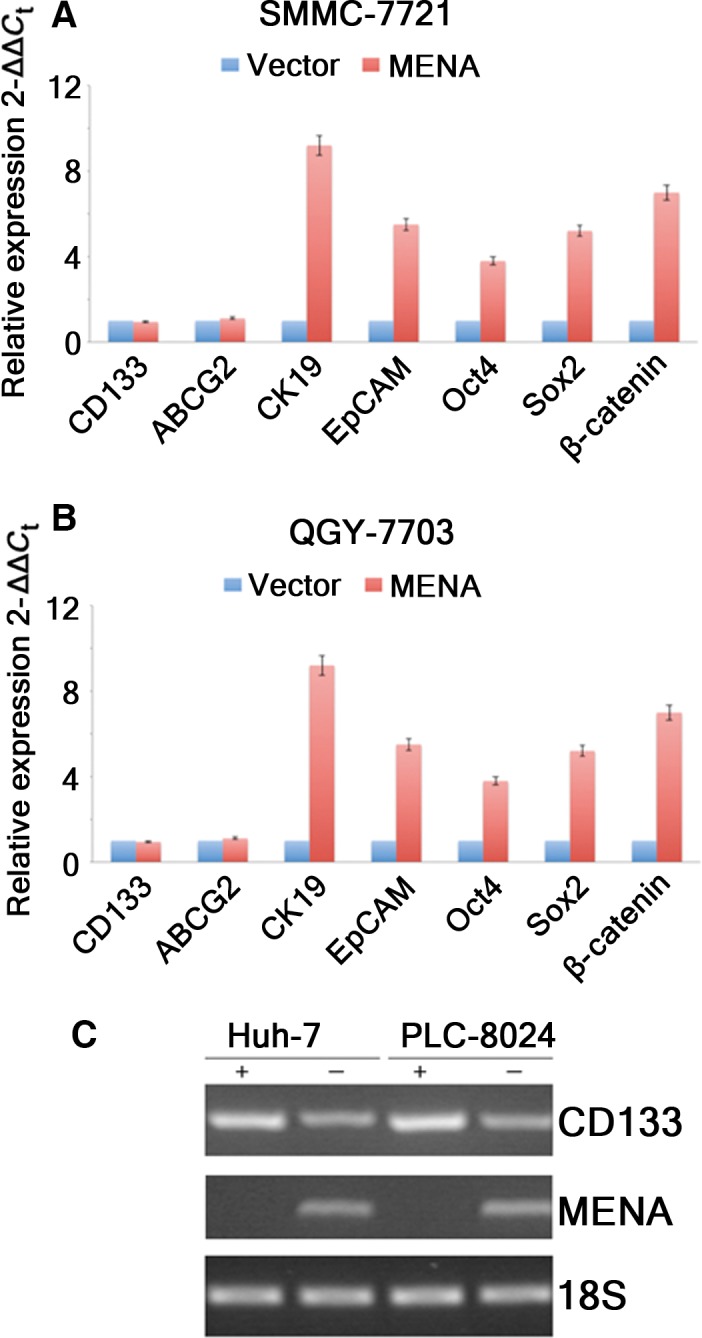
Overexpression of MENA upregulated hepatic progenitor/stem cell markers and Wnt/β‐catenin signaling markers in HCC cells. The mRNA levels of HPC markers CK19 and EpCAM, stem cell markers Oct4 and Sox2, and Wnt signaling marker β‐catenin in SMMC‐7721‐MENA (A) and QGY‐7703‐MENA (B) cells were compared with their vector control counterparts, respectively. (C) MENA expression levels were determined by RT‐PCR using RNA extract from CD133^+^/CD133^−^Huh7 and PLC‐8024 cells, respectively.

As enhanced tumorigenicity is a characteristic of CSCs, next we investigated whether overexpression of MENA impacted on CSC‐like characteristics of HCC cells. As shown in Fig. 3, overexpression of MENA dramatically upregulated the mRNA levels of hepatic progenitor cell (HPC) markers CK19 and EpCAM, stem cell markers Oct4 and Sox2, and Wnt signaling marker β‐catenin in both stable MENA HCC cell lines as compared with their vector control counterparts (Fig. 3A,B). However, the levels of CSC marker CD133 and stem cell marker ABCG2 did not change after overexpression of MENA.

Real‐time‐PCR analysis found that MENA expression was only detected in total RNA extract of CD133^+^ Huh7 and PLC‐8024 cells, but not in their CD133^−^ counterparts (Fig. 3C), indicating that only LCSCs express MENA. Taken together, these data suggested that MENA may play a role in CSC regulation of HCC cells.

### High MENA protein was associated with high mRNA levels of MENA, CD133, CK19, and EpCAM in human HCC tissues

**Figure 4:**
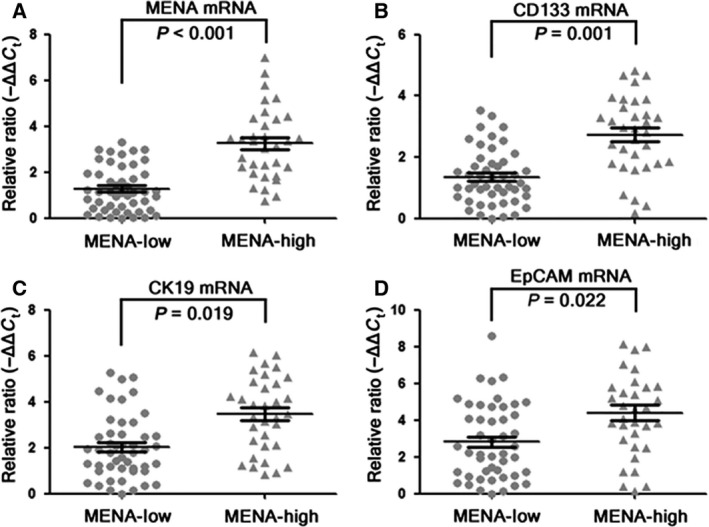
High MENA protein was associated with high mRNA levels of MENA, CD133, CK19, and EpCAM in human HCC tissues. Eighty‐one human HCC tissues were divided into MENA‐Low (*n* = 48) and MENA‐High groups (*n* = 33) according to their expression level of MENA protein. The mRNA levels of MENA (A), CSC marker CD133 (B), and HPC markers CK19 (C) and EpCAM (D) were determined by RT‐PCR and compared between two groups (Student's *t*‐test).

To further confirm whether MENA plays a role in CSC regulation of HCC cells, 81 human HCC tissues were divided into MENA‐Low (*n* = 48) and MENA‐High groups (*n* = 33) according to their expression level of MENA protein as previously described 26. The mRNA levels of MENA, CSC marker CD133, HPC markers CK19 and EpCAM were compared between two groups. As expected, MENA mRNA level was significantly higher in MENA‐High group than in MENA‐Low group (Fig. 4A). In addition, MENA‐High group had significantly higher mRNA levels of CD133 (Fig. 4B), CK19 (Fig. 4C), and EpCAM (Fig. 4D) as compared with MENA‐Low group. These data indicated that high expression of MENA was associated with elevated CSC markers in HCC cells.

### Overexpression of MENA promoted EMT, ERK phosphorylation, and β‐catenin protein level

**Figure 5:**
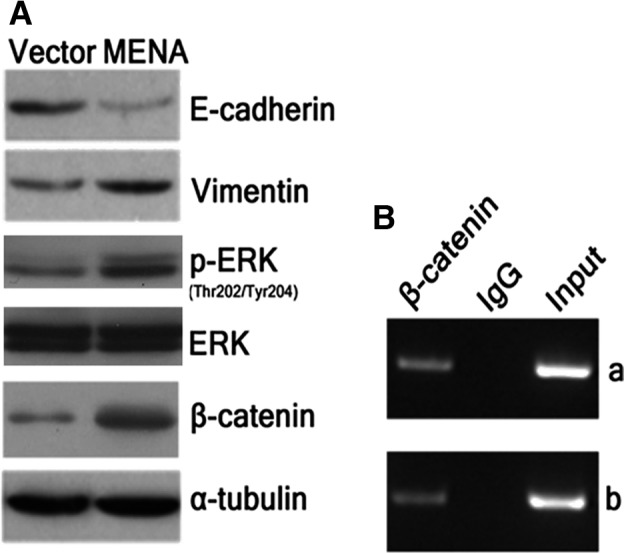
Overexpression of MENA promoted EMT, ERK phosphorylation and β‐catenin protein level. (A) The protein levels of E‐cadherin and vimentin, ERK, p‐ERK, and β‐catenin were determined by western blot and compared between QGY‐7703‐MENA and vector control cells. (B) ChIP was performed to analyze the β‐catenin protein binding to the promoter region of *MENA* gene in HCC cells. Mouse IgG was used as a control immunoprecipitate antibody, while DNA sample that has not been immunoprecipitated was used as an input control. Panel a and panel b were two replicates of the experiment. The results showed that β‐catenin directly bound with the promoter region of *MENA* gene.

Finally, we attempted to investigate the molecular mechanism underlying MENA‐mediated tumorigenicity enhancement in HCC cells. We examined the protein levels of two EMT biomarkers, E‐cadherin and vimentin. As shown in Fig. 5A, overexpression of MENA simultaneously downregulated E‐cadherin and upregulated vimentin in QGY‐7703‐MENA cells, suggesting that MENA promoted EMT. The data also showed that overexpression of MENA elevated protein level of p‐ERK (Fig. 5A), suggesting that overexpression of MENA activates ERK signaling pathway.

Previous study has demonstrated that MENA is a transcriptional target of Wnt/β‐catenin pathway 36. To address whether β‐catenin pathway is associated with MENA in HCC cells, we determined β‐catenin protein level in QGY‐7703‐MENA cells. The data showed that MENA‐overexpressing cells had a markedly increased protein level of β‐catenin as compared with vector control cells (Fig. 5A). The result of ChIP also confirmed that β‐catenin directly bound with the promoter region of *MENA* gene (Fig. 5B), demonstrating that MENA is a transcriptional target of β‐catenin in HCC cells. These data implied that EMT, ERK, and β‐catenin signaling pathways may be involved in the mechanism underlying MENA‐mediated tumorigenicity enhancement in HCC cells.

## Discussion

In this study, we investigated the oncogenic potential of MENA and its underlying mechanism in HCC cells. The data showed that overexpression of MENA significantly enhanced colony‐forming and migration abilities in HCC cells. Overexpression of MENA in HCC cell lines also upregulated the HPC markers (CK19, EpCAM) and the stem cell markers (Oct4, Sox2). In addition, high MENA protein level was associated with elevated mRNA levels of the hepatic progenitor/stem cell markers CD133, CK19, and EpCAM in human HCC tissue samples. Furthermore, overexpression of MENA promoted EMT and ERK phosphorylation and level of β‐catenin in HCC cells. These data suggested that MENA has the ability to enhance tumorigenicity and the expressions of CSC and EMT markers in HCC cells, which may be involved, at least partially, in the ERK and Wnt/β‐catenin signaling pathways.

Our previous work showed that high expression of MENA in human HCC tissues is associated with poor cellular differentiation 26, implying that MENA may play a role in the dedifferentiation of HCC. The current study further demonstrated that HCC samples with elevated MENA protein displayed high levels of LCSC markers CD133, CK19, and EpCAM. In addition, MENA‐overexpressing HCC cells showed enhanced hepatic progenitor/stem cell markers. CD133 is an important CSC surface marker in various tissues including HCC. Previous study reported that CD133^+^ cells accounted for 65% of Huh7 cells 37. CD133^+^ cells had a greater colony‐forming efficiency, higher proliferative capacity, and greater tumorigenicity 38. In addition, CD133^+^ HCC cells have been shown to be more resistant to chemotherapy and radiotherapy through activation of the Akt/PKB and Bcl‐2 survival pathways 37, 39, 40, 41. Chen *et al*. 42 showed that CD133^+^EpCAM^+^ Huh7 cells possessed elevated capacities for colony formation, differentiation, chemoresistance, and tumorigenicity in mice. Our data showed that MENA expression was detected only in CD133^+^ HCC cell lines Huh7 and PLC‐8024 cells, but not in their CD133‐ counterparts. These results suggested that there is an association between CD133 and MENA. The phenomenon that overexpression of MENA in HCC cell lines resulted in unchanged level of CSC marker CD133 may indicate that MENA may be a downstream protein of CD133 so that overexpression of MENA had no effect on CD133 level. Further study is required to elucidate the relationship between CD133 and MENA in CSCs. These results also indicated that MENA may play a role in the regulation of CSC in HCC cells. It is worth to further investigate whether MENA regulates CD133^+^ LCSCs by establishing a MENA‐overexpressing CD133^+^ HCC cell model.

In the current study, MENA‐overexpressing HCC cells presented reduced E‐cadherin and elevated vimentin proteins, indicating that the cells underwent EMT transformation. The EMT transition plays a crucial role in cancer invasion, metastasis, as well as for therapeutic resistance. CSCs have the capacity of self‐renewal, migration, and invasion and are resistant to radiation and chemotherapy. Therefore, metastatic cancer cells may simultaneously possess the characteristics of both EMT and CSCs 43. Accumulating evidences have identified the correlations between EMT and the CSC transformation in cancer cells 44, 45. Mani *et al*. 45 reported that immortalized human mammary epithelial cells undergoing EMT induce the expression of stem cell markers and form mammospheres, soft agar colonies, and tumors. In addition, several studies have suggested that HCC cells can acquire CSC‐like characteristics through the EMT transition, exhibiting metastasis and therapeutic resistance 46, 47, 48. Thus, both CSC and EMT are important for the invasion and metastasis of HCC. Our previous finding demonstrated that high expression of MENA in human HCC tissues is associated with advanced tumor stage 26. In this study, MENA‐overexpressing HCC cells have elevated expression levels of both CSC and EMT markers and enhanced capacities of migration and colony formation, suggesting an oncogenic potential of MENA in HCC. In addition, our observations further supported the correlations between EMT and the CSC in HCC. To our best knowledge, our data report the effects of MENA on expressions of CSC and EMT markers in HCC cells for the first time, which also have not been described in any other cancers.

Both the molecular mechanisms of EMT and CSC are complicated, and there are many common regulation mechanisms between these two procedures. ERK and Wnt/β‐catenin signaling pathways have been shown to be involved in the regulation of both stemness phenotypes 31, 33 and EMT 32, 34 in HCC. Our data showed that both the protein levels of p‐ERK and β‐catenin elevated in MENA‐overexpressing HCC cells. In addition, the ChIP data showed that MENA is a transcriptional target of β‐catenin in HCC cells. These findings suggested that MENA may regulate CSC features and EMT in HCC through Wnt/β‐catenin and ERK pathways. Further study is needed to verify the detailed mechanism.

Several limitations should be mentioned in the current study. First, although our results found that overexpression of MENA elevated the levels of several LCSC biomarkers in HCC cells, we did not examine the effect of stable knockdown of MENA on the levels of LCSC biomarkers in high MENA‐expressing cells. We also did not evaluate the effects of MENA overexpression on the other key features of CSC, such as capacity of self‐renewal, drug resistance (i.e., sorafenib), and sphere formation to further confirm the CSC regulation function of MENA. In addition, we did not simultaneously implement the results for another MENA‐overexpressing cell (SMMC‐7721‐MENA cell) in this study, and the immunofluorescence data of colocalization of MENA and stem cell markers (CK19 and CD133) in human HCC tissues are important to validate MENA as a stemness marker. Furthermore, in the following study, it is necessary to isolate MENA^+^ LCSCs from fresh HCC surgical samples. These cells should be characterized for stemness features and proliferation ability using both *in vitro* and *in vivo* models. All these limitations should be addressed in the following study.

In summary, this study demonstrated that overexpression of MENA enhanced expression of CSC markers and EMT markers, as well as the tumorigenicity in HCC cells, which provide partial mechanisms for the oncogenic role of MENA in the metastasis and recurrence of HCC. Our findings may be helpful for the development of new therapeutic strategies for HCC.

## Author contributions

KH and BL planned experiments; PH performed experiments; HL, ZY, QW, and ZX analyzed data; JL, HH, SX, and PZ contributed reagents or other essential materials; KH and PH wrote the manuscript.
